# GIS-Based Tool for Pest Specific Area-Wide Planning of Crop Rotation Distance with Land Use Data

**DOI:** 10.3390/insects15040249

**Published:** 2024-04-04

**Authors:** Manuela Schieler, Natalia Riemer, Paolo Racca, Benno Kleinhenz, Helmut Saucke, Michael Veith, Bernd Meese

**Affiliations:** 1 Central Institute for Decision Support Systems in Crop Protection, Rüdesheimer Str. 60-68, D-55545 Bad Kreuznach, Germany; racca@zepp.info (P.R.); kleinhenz@zepp.info (B.K.);; 2 Department of Biogeography, Faculty of Regional and Environmental Sciences, Trier University, Universitätsring 15, D-54286 Trier, Germany; veith@uni-trier.de; 3 Section of Ecological Plant Protection, Faculty of Organic Agricultural Sciences, University of Kassel, Nordbahnhofstr. 1a, D-37213 Witzenhausen, Germany; natalia.riemer@uni-kassel.de (N.R.); hsaucke@uni-kassel.de (H.S.)

**Keywords:** area-wide pest management, crop rotation distance, integrated pest management, Python script, *Cydia nigricana*

## Abstract

**Simple Summary:**

Today’s society demands a better balance between the needs of agricultural production and environmental protection, particularly in reducing the application of pesticides. Compared to the past, fewer pesticide active ingredients are allowed and available in the European Union. One pest that is controlled by pesticides is our model pest, the pea moth (*Cydia nigricana*), which can cause damage to pea seeds (*Pisum sativum*). The aim of this study was to develop alternative and preventive strategies that can be used as crop protection in the shape of a GIS-based planning tool for crop rotation with a minimum distance to avoid specific pests. In our tool, the distance between current and previous pea sites was important, because insect pests follow their host plants. The hypothesis is that the greater the distance, the less infestation. We developed a web tool to create buffers with different distances around the previous pea sites to calculate the infestation risk for future pea sites. The web tool can be adapted in terms of distance and risk classes for other pests. Consequently, our tool helps to avoid pest infestation, and therefore, farmers can reduce pesticide applications, which protects the environment and strengthens biodiversity.

**Abstract:**

Crop rotation is an important strategy for pest reduction. For mono-, or oligophagous pests that overwinter at a previously infested site, crop rotation means that the pests must find new host crop sites in the following year, and it is more efficient if a pest-specific distance is applied. Here, we report the development of a GIS-based tool for efficient cultivation planning using the example of the pest complex pea moth (*Cydia nigricana*) and grain and green peas (*Pisum sativum*). Monitoring data for four consecutive years (2016–2019) from 513 sites were used. Infestation of pea seeds and the distance to the previous year’s pea sites were recorded. An adjustable Python script was developed by means of infestation–distance–correlation as a pest and crop-specific minimum migration distance (MD). The output of the tool is a risk map as decision support for cultivation planning. It shows different risk buffers with distances from 1261 m to 1825 m, depending on the cultivation type. The web tool is easily adjustable to other pests and crops anywhere in the world. The tool helps to prevent damages caused by agricultural, mono-, or oligophagous insect pests and consequently reduces pesticide applications for the benefit of the environment and biodiversity.

## 1. Introduction

Crop rotation is a proven and valuable technique for ecosystems and the reduction of soil-borne pests and diseases [1,2,3,4]. For sustainable crop protection, it is better to avoid pests in advance than the obligation to have to control them with pesticides. For example, Wright [2] studied crop rotation effects on Colorado potato beetle (*Leptinotarsa decemlineata*) infestation and found out that crop rotation is the best way to control pest species with few hosts and little migration distances. Area-wide crop rotation has an even greater effect on pest populations.

Further reasons for crop rotation are limited approaches for pest control in organic farming, the availability of allowed pesticide active ingredients, insecticide resistances, and a lack of specific pesticides against target species. First, in organic farming, the efficacy of organic pesticides is often lower compared to conventional pesticides [5]. Second, fewer pesticide active ingredients are allowed to be used in the European Union, pesticides like some neonicotinoids can no longer be used even though they have been effective in insect pest control [6]. The third important factor, insecticide resistance, makes it harder to control some pest species. For Colorado potato beetles, insecticide resistance is well known for neonicotinoids [7,8], pyrethroids, nereistoxins [9], and novaluron [10]. The fourth reason is that there are some new pest species, for example, the cixiid planthopper *Pentastiridius leporinus*, without pesticide control strategies so far. Furthermore, there is the “National action plan for a sustainable application of pesticides” in Germany [11], which claims to use pesticides responsibly, meaning not only to protect crop plants but also to protect human and animal health. In summary, there are many reasons to concentrate on crop rotation distance as an avoidance strategy for insect pests.

There are pests that overwinter site-specifically or at least in close proximity to previous host plant sites from where they have to migrate to find their host in the next season. The pest-specific distances, which they have to overcome to reach the new host plants, are important for a more efficient crop rotation strategy. Furthermore, there are several studies on crop rotation distance of Colorado potato beetles [12,13,14,15,16], which cause damage by defoliating potato plants [7]. For example, with a distance > 100 m, the infestation risk is reduced [16], and with a distance > 400 m, the effect is even better [14,16,17]. Additionally, with a rotation distance of 300–900 m, the insecticide necessity was reduced by 50% versus a non-rotated field [12]. Consequently, crop rotation will be more efficient with distances adapted to the pests and digitalized procedures, like a web tool. Therefore, it is necessary to research pest-specific crop rotation distances [15,17] for pests that overwinter site-specifically in harvested sites or at least close to them, as well.

The use of geographic information systems (GIS) within decision support systems (DSS) in agriculture can be very effective and useful, and many different applications already exist [18,19]. If input data are available, they are area-wide applicable. When developing a web tool, using a Python script is convenient, because it is easily adjustable with respect to crop rotation distances and different pests as soon as needed parameters are available. Additionally, automated calculations save a lot of time. For Colorado potato beetles, Weisz et al. [13] developed models to calculate infestation as a function of migratory distance. With one of the models, they created a risk map for planned potato fields, as an interpolated heat map, which shows the infestation risk compared to a non-rotated field. Thus, a risk map according to the crop rotation distance could reduce or avoid infestation by pests in advance.

In our study, we used the pea moth (*Cydia nigricana,* Lepidoptera: Tortricidae) as a model species. Pea moths can cause great damage to pea seeds (*Pisum sativum*, Fabaceae), which leads to yield and quality loss [20,21]. The model species overwinters as the fifth instar in the soil of previous pea sites. In spring, the adults emerge and search for new pea sites, lured by the odor of pea flowers [22]. Former studies focused on the minimum crop rotation distance (MD) of pea moths, which describes the distance between infested pea sites of the last year and the ones of the current season. For example, Thöming et al. [23] discovered that an MD of 500 m for green peas had a positive effect on a decreasing infestation by pea moths. Huusela-Veistola and Jauhiainen [24] found that seed and pod infestation decreased exponentially with the distance to a previous grain pea field of >1000–3000 m, and similar results are shown by Riemer et al. [25].

We show how to prevent damage to crops by insect pests that overwinter site-specifically by introducing a planning tool as an area-wide application, which was developed by the Central Institute for Decision Support Systems in Crop Protection (German acronym: ZEPP) as part of the project CYDNIGPRO (CYDia NIGricana PROgnosis). The infestation of pea fields by pea moths as well as the minimum distance to previously infested pea fields were analyzed and implemented in a risk map in order to reduce and avoid damage to peas. The results of the project are partly used as input for a risk map for improved crop rotation distances. Such a risk map has not been established for practical use in Germany so far. The planning tool can be used and adapted to other pests worldwide if input data are available.

## 2. Materials and Methods

**Figure 1:**
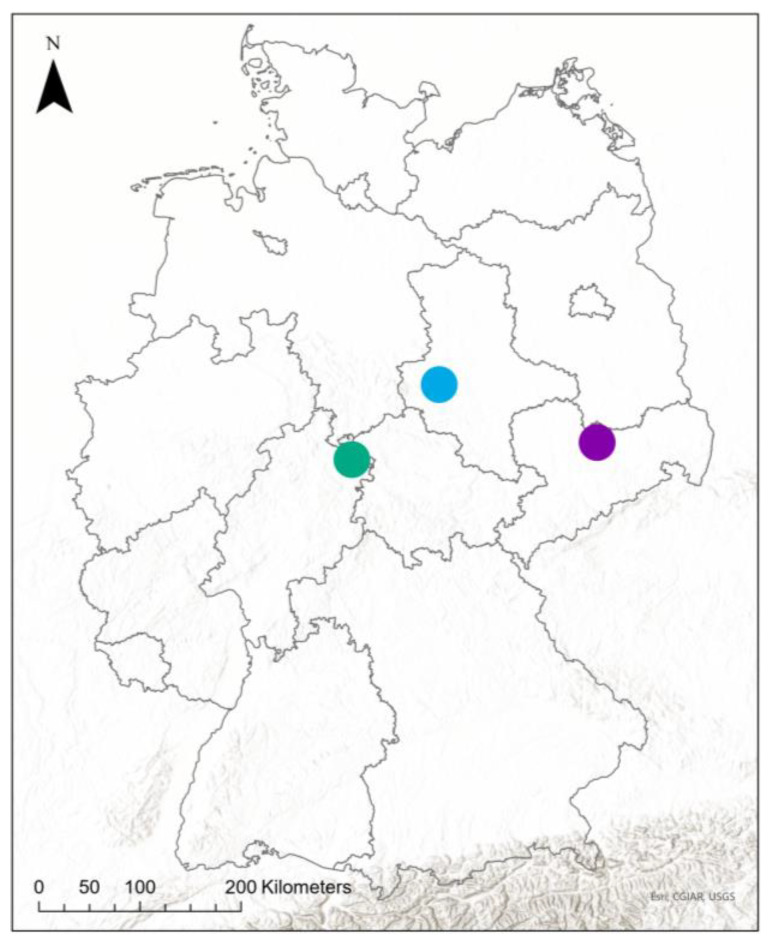
Map of Germany with the location of the model areas, ST = blue, SN = purple, HE = green (map source: Esri, CGIAR, USGS).

Field data for the development of the risk map were acquired between 2016 and 2019 in three representative German pea-growing areas, which are located in the federal states Saxony-Anhalt (ST), Saxony (SN), and Hesse (HE) (Table 1). In Saxony-Anhalt, northeast of the Harz Mountains, in the proximity of Quedlinburg (51.93–51.74 N, 10.98–11.29 E, WGS84, Figure 1), the cultivation of peas has a long history, especially for seed production. The “Lommatzscher Pflege” (51.40–51.18 N, 13.20–13.53 E) in Saxony is a traditional green pea-growing area, a major one for frozen green pea products in Germany. The cultivation of peas in Hesse (51.38–51.14 N, 9.75–10.12 E) is not as intense and commercial as in Saxony-Anhalt and Saxony, the pea sites are much smaller, and most of the peas are forage crops. Each model area had a diameter of approx. 30 km. The sample sites were cultivated by local farmers or agricultural cooperatives. Some of the farmers used insecticides, others did not. The data were used in three different groups: all grain peas, and grain peas with and without insecticide treatment. For the outcome, the data grain peas with insecticide treatment are not usable, because the goal is to use satellite data, with which those differences are not detectable.

### 2.1. Data Acquisition

#### 2.1.1. Infestation Data

To determine the correlation between infestation and minimum crop rotation distance, the sampling of the infestation was required. Therefore, at the end of the growing season, at each pea site, a sample of 100 pods was taken to record percentage infestation by pea moths that is easily recognizable from nutritional traces, mostly around the seeds. We collected the pods along the tractor tracks, starting after the headland, for approx. 50 m. The number of seeds infested by pea moths was counted and converted in percentage.

#### 2.1.2. Pea Site Locations

**Figure 2:**
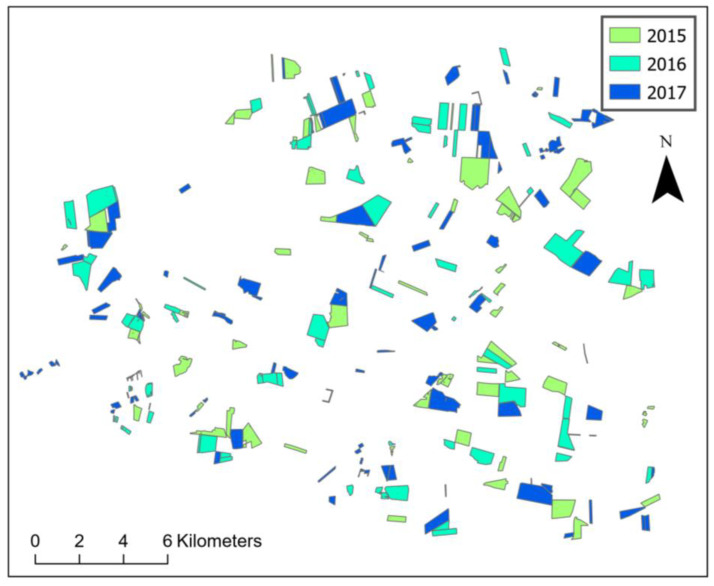
Map of pea sites in model area ST for the years 2015–2017 as an example. Current pea sites and those of the previous year are often right next to each other, see the appendix for SN and HE.

**Figure A1:**
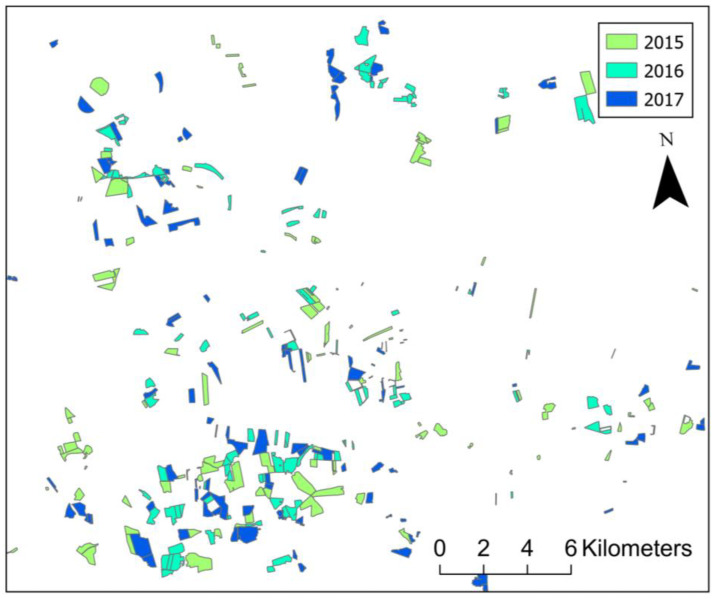
Map of pea sites in model area SN for the years 2015–2017. Current pea sites and those of the previous year are often right next to each other.

**Figure A2:**
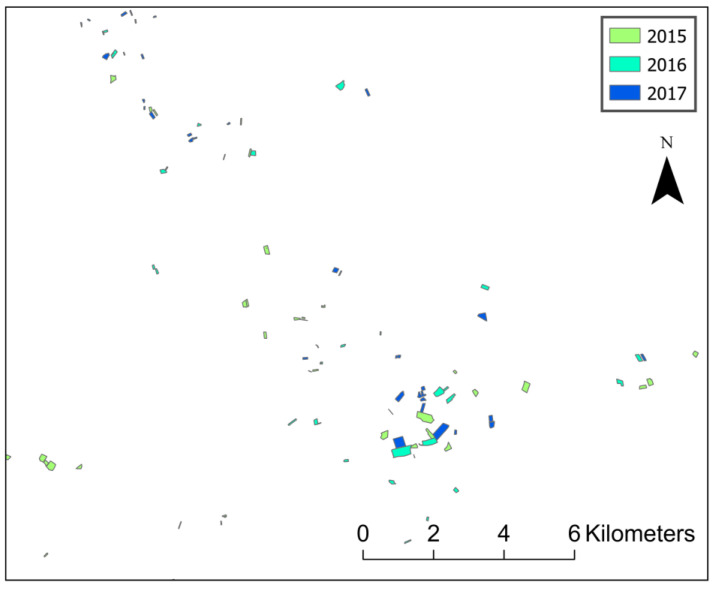
Map of pea sites in model area HE for the years 2015–2017. Current pea sites and those of the previous year are often right next to each other.

There are different possibilities to obtain information about the location of pea sites. Surveying farmers in a greater area is too labor-intensive. Consequently, a larger dimension to obtain area-wide locations of pea sites is needed. In Germany, one possible format is anonymous InVeKoS (Integriertes Verwaltungs- und Kontrollsystem) data. These data are available in a vector format (polygon shapefiles) and are provided by governmental agencies that are responsible for agricultural subsidies related to the common agricultural policy (CAP) of the EU (Figure 2, Appendix A: Figure A1 and Figure A2). Examples of these programs are “ecological focus areas” or “crop diversification” within the greening direct payments [26,27]. In our study, these geographical locations were used as sample sites and for all further calculations. The data we used were from the years 2015–2019, whereas the data from the year 2015 only served as previous pea site locations, not as sample sites. In the maps of the model areas, we show the examples for the years 2015–2017 to obtain an overview of how close the pea sites are from one to the next year.

### 2.2. Infestation–Distance–Correlation

In spring, pea moths that had overwintered in pea sites have to overcome the distances to new sites with their host plants. Therefore, we correlated the distance and infestation data to provide basic information about the minimum crop rotation distance (MD). We used the provided polygon shapefiles to calculate the distance between the current pea sites and those of the previous year, from field edge to field edge. Only the nearest pea site was used. For the calculation of infestation as a function of MD, we used the exponential decay function of Weisz et al. [12], Thöming et al. [23] and Huusela-Veistola and Jauhiainen [24]:

(1) $$y=a\times e^{\left(-\frac{\mathrm{MD}}{b}\right)}$$

where a is the y-intercept, b is the inflection point of the slope, MD is the minimum distance in meters to the closest pea field of the previous year, and y is the estimated infestation in percent. The correlation of infestation data and minimum distance to the closest previous pea site serve as background information for the risk classes provided by the risk maps as described in the next section. The influence of multiple previous pea fields is explained in section “overlapping buffer zones”.

### 2.3. Geographical Implications

Crop site locations of the previous year are the base of the calculation. Only the fact that there used to be a cultivated site is taken as information, not the absolute infestation of each site, due to the effort to gather the infestation data of each site each year. The calculations were conducted with the ArcGIS Pro 3.1.0 (Esri) and Python 3.6.

#### 2.3.1. Python Script

**Figure 3:**
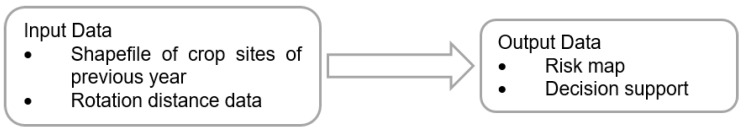
Workflow of the Python script.

We provide a universal script for crop rotation options for different crops. Therefore, several parameters are adjustable, such as distance, the number of risk classes, and the value of the risk classes. The risk classes can be linear, exponential, or individually adapted. In this paper, the proceedings are presented using the example of pea moths and our study data. In Figure 3, the general workflow of the Python script is visualized.

#### 2.3.2. Buffer Zones

The first step is to build buffer zones with different distances. They are calculated around the input polygons, which are the crop sites of the previous year. It is possible to create one or more buffer zones with desirable distances depending on the pest species data. In case of more than one buffer zone, there is always a ring-shaped buffer built around the next inner buffer.

#### 2.3.3. Risk Classes

Each buffer zone gets a specific risk class. In case of more than one buffer zone, risk classes decrease with an increase in distance to previous year’s crops. Risk classes may have linear or exponential numerical order, be in percent, or other individual classes may be used.

#### 2.3.4. Overlapping Buffer Zones

**Figure 4:**
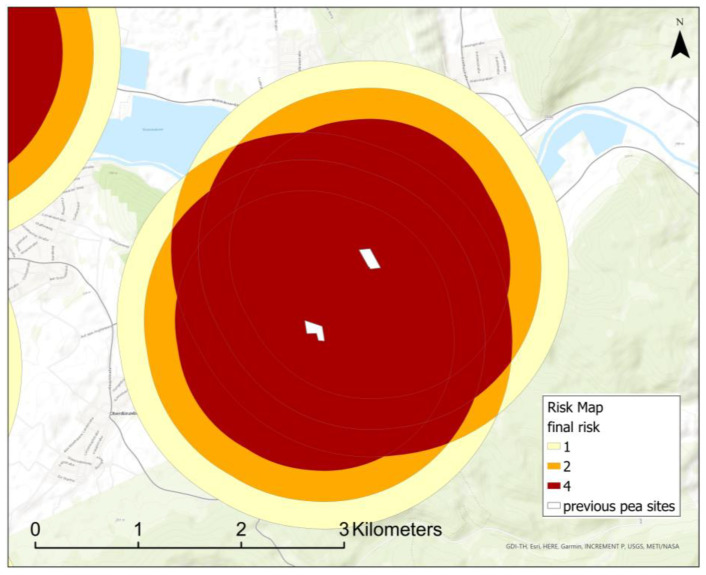
Risk classes of overlapping areas are automatically raised to the next highest risk class (here exponential). In this case, the risk comes from two pea sites of the previous year: white polygons (map source: GDI-TH, Esri, HERE, Garmin, INCREMENT P: USGS, METI/NASA).

The next step unites overlapping buffer zones. If two or more previous fields are close to each other, buffer zones may overlap. In this case, the overlapping layers are automatically deleted except for one. The polygon that is left is automatically set to the next highest risk class because sources of infestation come from at least two directions (Figure 4).

**Figure 5:**
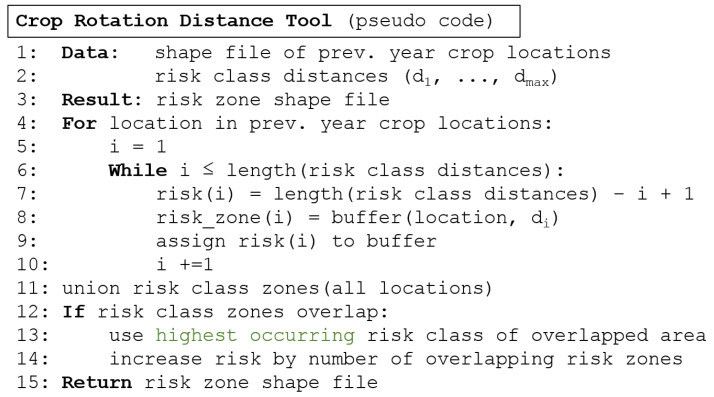
Pseudo code of crop rotation distance tool.

In Figure 5, a pseudo code of the crop rotation distance tool is written. It shows the stylized single steps of the calculation, which are also described above.

## 3. Results

### 3.1. Infestation–Distance–Correlation

**Figure 6:**
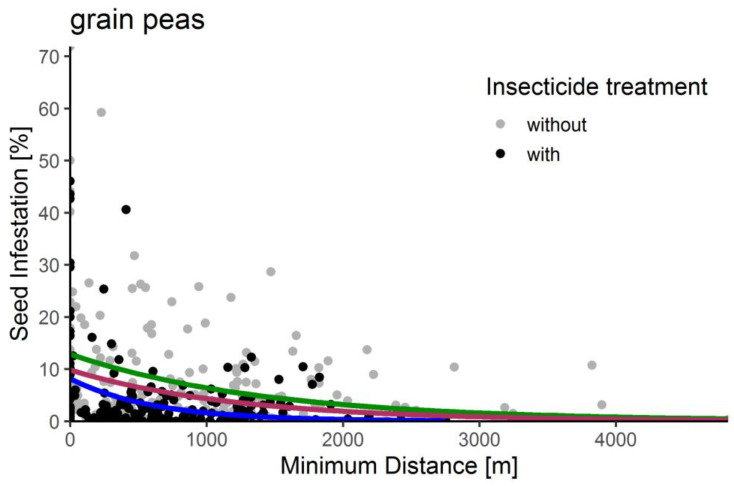
Seed infestation in percent as a function of minimum distance in meters. Each dot depicts one sample site. The gray dots symbolize the sites without insecticide treatment, and the black dots are the ones with insecticide treatment. The red curve shows the exponential decay function of all grain peas, the green curve indicates sites without insecticide treatment (n = 188), and the blue curve indicates sites with insecticide treatment (n = 182).

In the following, the result of the infestation–distance–correlation is described, which results in the recommended crop rotation distances. In Figure 6, seed infestation is plotted against MD. The observed data are shown with the fitted model curves for all grain peas (red), grain peas without insecticide treatment (green), and grain peas with insecticide treatment (blue). For example, for all grain peas (red), the infestation rate is 9.84% at 0 m, declining to 4.32% at 1000 m and 2.86% at 1500 m; the latter is less than a third of the infestation rate at 0 m.

In Table 2, the parameters of the model equations are described. For all grain peas, the effective crop rotation distance is 1261 m ± 299 m, with an RMSE goodness of fit parameter of the exponential decay function of 9.1. Additionally, the calculations were also conducted for grain peas with and without insecticide treatment. For grain peas with insecticide treatment, the effective crop rotation distance is 619 m ± 187 m, with an RMSE of 7.41 and an R^2^ of 0.07. For grain peas without insecticides, the effective crop rotation distance is 1426 m ± 399 m, with an RMSE of 9.98 and an R^2^ of 0.08. The poor R² of all three calculations reflects the wide variation in the data in Figure 6. However, the significant estimates prove that the tendency of the curves is significant. Consequently, we decided to offer the following risk buffers:

- 1261 m = inner risk buffer, which is parameter b for all grain peas;
- 1560 m = middle risk buffer, which is parameter b for all grain peas plus standard error;
- 1825 m = outer risk buffer, which is parameter b for grain peas without insecticides plus standard error.

The decision on which risk buffer should be used depends on the cultivation type, such as organic or conventional, green or grain peas. Nevertheless, we highly recommend using at least the inner safety buffer and not parameter b of grain peas with insecticides, since we do not know if a previous pea site was treated or not. In the case of organic pea cultivation, we recommend using the outer risk buffer.

The same calculation was not possible for green peas because the mean infestation was very low (0.11%); therefore, we decided to use a safety buffer of 500 m as Thöming et al. [23] recommended.

### 3.2. Geographical Implication

#### 3.2.1. Risk Map

**Figure 7:**
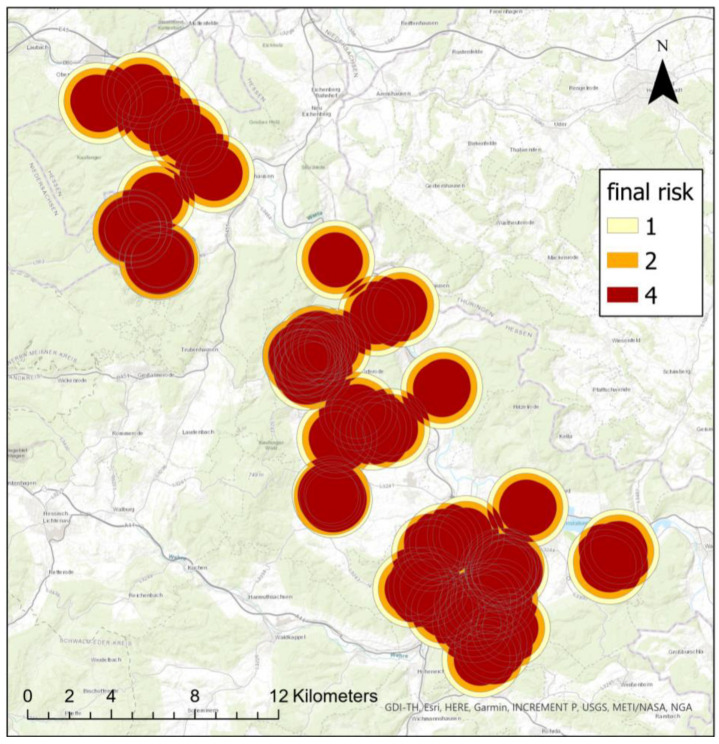
Risk map with buffers around potential infestation sites of the previous year (map source: GDI-TH, Esri, HERE, Garmin, INCREMENT P: USGS, METI/NASA, NGA).

The first part of the calculations results in a risk map, which shows the buffer zones according to risk classes around the crop sites of the previous year (Figure 7). If the infestation risk comes from multiple directions, buffer zones overlap and risk classes are increased to the next highest class, here exponential from two to four.

#### 3.2.2. Planning Tool

**Figure 8:**
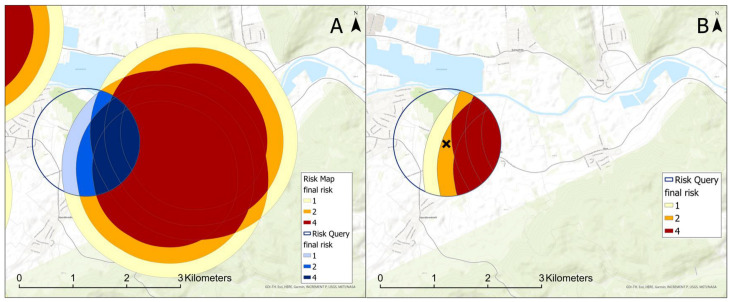
(**A**): For the user’s query (blue), a buffer around geographical coordinates is clipped out of the risk map. (**B**): The displayed user output is the clipped region of interest, the ‘×’ marks the queried geographical coordinates. The chosen new pea site has a risk potential, apparently coming from two previous pea sites (map source: GDI-TH, Esri, HERE, Garmin, INCREMENT P: USGS, METI/NASA).

In the second part of the calculations, the user defines the new pea site of interest (risk query). The planning tool then cuts out a circular zone around the queried coordinates (Figure 8A, blue circle) and provides it as the site-specific risk map (Figure 8B). The user can now decide if the planned site is suitable or not. In addition, it is possible to identify directions of risk sources. If there is an option to grow peas on an alternative site, the user can now choose a site in a less risky area.

## 4. Discussion

We here show how to mitigate crop damage by site-specifically overwintering insect pests and how this information can be implemented in an area-wide, commonly available web tool. We digitalized and automatized the calculation of infestation risk as a function of minimum crop rotation distance (MD). A key factor for obtaining effective pest reduction is the pest-specific MD.

### 4.1. Infestation–Distance–Correlation

The infestation–distance–correlation showed that the greater the distance to the previous year’s pea site, the less infestation by pea moths (Figure 6, Table 2). For grain peas, the effect was apparent, and the effective rotation distance for all grain peas is 1261 m ± 299 m. We used these distances for the inner (1261 m) and the middle risk buffer (1560 m). The outer risk buffer (1825 m), which is the worst-case scenario for grain peas without insecticides, should be used if organic peas are grown or if it is known that the previous year’s infestation was very high. It cannot be recommended to use a smaller buffer, since with the use of satellite data, we do not know if a previous year’s pea site was treated with insecticides or not. Even though the function almost seems to be linear within the examined range, the underlying function has been proven to be an exponential decay function by Huusela-Veistola and Jauhiainen [24], Thöming et al. [23], Weisz et al. [12], and Riemer et al. [25] in their studies. The MD for grain peas of 1–3 km was effective in reducing the risk of pea moth infestation, depending on the year and cropping system [24]. They recommend a distance of 1.5 km to previous pea fields, which is similar to our recommendation. As a consequence of the calculation, insecticides could be reduced, in terms of fewer applications, or left out, if the determined distance to the previous year’s crops is maintained.

Green peas, which are harvested around BBCH 79 in a green, immature, and tender condition, do not implicate a big risk for infestation in the following year. Most of the pea moths are not able to complete their life cycle, due to early harvest. Hence, the infestation pressure in dense and intensive green pea cultivation areas does not get really high. Therefore, we decided to use a comparably small risk buffer of 500 m around the previous year’s green pea sites. In organic green peas, an MD of 500 m resulted in a significant reduction in larval infestation [23]. 

Similar studies concerning insect pests have been conducted in the past. For example, an MD of more than 400 m was effective in reducing Colorado potato beetle infestation in the following year [12,14,16], with increasing distances further reducing infestation. Carrière et al. [28] studied *Lygus hesperus*, the western tarnished plant bug, and its maximum migration distance. They found that at a maximum distance of more than 1500 m, there is no additional spread of *L. hesperus* from alfalfa into cotton, which is important for farmers.

### 4.2. Geographical Calculations

**Figure A3:**
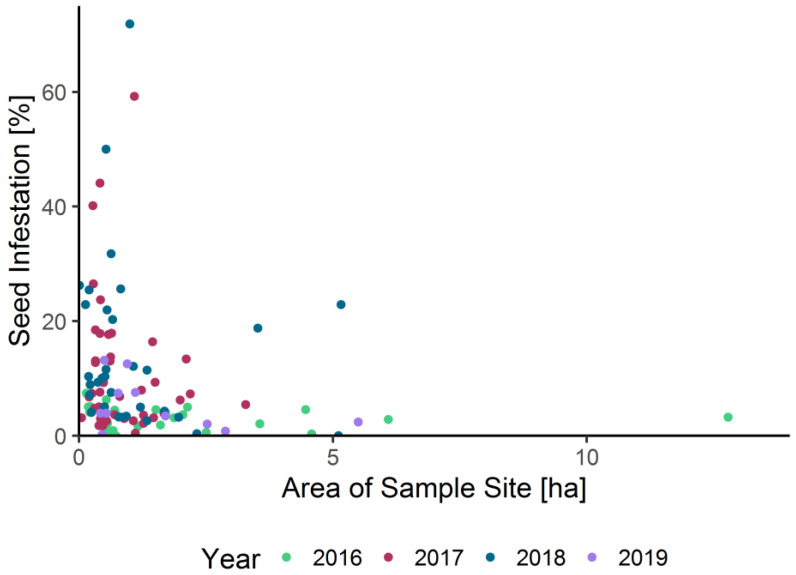
Seed infestation in percent as a function of sample site area in hectares. In the example are sites from Hesse that were not sprayed with insecticides. Small sites can have a high and low infestation by pea moths; R² = 0.02, linear regression. Peas that are cultivated for seed production must have a very low infestation level for seed approval.

Our new GIS-based web tool generates a risk map and provides decision support for applicants (Figure 8). Although only the nearest pea site was used as input for the infestation–distance–correlation, the risk map calculations include all previous pea sites in the vicinity of a potential new site by overlapping risk buffers (Figure 4). Interestingly, we found no correlation between infestation intensity and the sizes of pea sites (Appendix A, Figure A3). While one might expect a higher infestation probability with increasing site sizes due to a more intense flower odor, in our study we found numerous small sites with high infestations as well as large sites with low infestation. Therefore, we assume that the number and the distance of fields in the vicinity are the main factors that affect infestation in the following year.

In the past, different approaches to mitigate infestation by insect pests were developed. For example, Weisz et al. [13] developed an “all source model” for Colorado potato beetles by calculating one risk zone or interpolated hotspots with different risk classes for an area, so the new fields can be placed outside the risk zone. In our risk map, there are risk buffers around each previous field, which means that the provided risk areas are much more differentiated. Beckler et al. [29] also created an interpolated abundance map, which shows hot spots of corn rootworms (*Diabrotica barberi* and *Diabrotica virgifera virgifera*) emergence. Compared to our approach, both approaches require annual area-wide monitoring, so to reduce the labor required, we decided to use the location of the pea sites as the main input factor.

In the future, continuous annual input data will be required for each crop. In this regard, the imminent availability of processed satellite data will be a huge enhancement. Waldhoff et al. [30] classified major crops to analyze crop rotation sequences in the Rur catchment in Germany. Meroni et al. [31] also classified major crops or crop groups and their phenology as BBCH stages all over Europe [32]. By using phenological timelines, it can be easier to classify the different pea-growing types, like grain peas, green peas, and green peas for propagation. Maps can be made available by different providers (e.g., www.map.onesoil.ai, [33]) by the classification of satellite images during vegetation periods, however by now mostly for main cultures such as wheat, corn, and rape seed or combined groups such as vegetables. Some German research institutes are still in the development phase to create maps with classified crops for permanent use, such as Thünen Institute [34], and FERN.Lab with Habitat Sampler and Minimal Sample Classifier (MiSa.C) [35,36]. The advantage of classified satellite images is that information on cultivated crops is area-wide, freely available, and does not depend on the application for agricultural subsidies. However, long-lasting cloud cover or difficult-to-distinguish combined cultures can pose a problem regarding these not-yet-available data sets.

### 4.3. Limitations

Parameters such as soil type, soil temperature, and parasitoids—currently not included in our model—may significantly affect infestation probability and overwintering. Finding out all relevant site effects might improve further infestation predictions but was not within the scope of this study. In our study, only the parameters distance and infestation were included. Nevertheless, other landscape structures such as urban areas and forests can serve as barriers. Since we do not have any study results for our model pest, we were not able to include such landscape structures in our calculations.

In some cases, the infestation can be high despite assumed large distances due to the federal borders, because InVeKoS data were only available for the federal states with model areas. Moreover, InVeKoS data are not in all cases crop-specifically classified, especially for our use. For example, a site might be labeled as bee pasture, but actually, it can be a pure pea site or include pea plants. Ecological focus areas like bee pastures could not be taken into account in our calculations.

Our tool cannot be used for polyphagous insects, such as some aphids, especially *Myzus persicae*, because it is impossible to detect their primary host *Prunus* spp. (holocycle) as well as all of the secondary hosts (anholocycle) with satellite data. Insects that change their host multiple times during the year are not suitable for the tool because the geographic position of the previous source is unknown. In addition, there are insects, here also aphis, for example, that can be spread by wind because of their light weight as aerial plankton. So, the real source of these aphids is in both cases not detectable.

### 4.4. Outlook

We here used pea moths and pea crops in three German growing areas as a model system to develop a GIS-based planning tool for crop rotation distance in order to minimize pea moth infestation rates. However, the tool can easily be adjusted to other insect pests, such as Colorado potato beetles on potatoes, Cixiid planthoppers on sugar beets, and corn rootworms on corn. Also, infestation with other pests such as fungi, nematodes, or pathogens can survive in the soil for several years, so in the next step numerous different pre-crops could be modeled. When more than one pre-crop are host plants for pests, they can be included. In addition, pest species-specific dispersion barriers can be implemented.

The use of crop rotation distance to ensure proper buffers between crops planted in one year and the next is best implemented through a system of area-wide management. Wright [2], Weisz et al. [13], and Sexson and Wyman [14] already recommended rotating crops through the cooperation of large groups of farmers in an area to ensure that host crops are not planted in high-risk areas. The goal should be to digitalize the cooperation of farmers for better area-wide communication and area-wide pest management.

Currently, our tool is only available for registered members on the Internet platform www.isip.de (information system for integrated plant production, [37]), a plant protection service provider that can be used by advisors and farmers in Germany. By the time of publication, the tool is completely programmed and implemented in the platform with an interim solution, where users must provide pea sites of the previous year manually, at least until satellite data are available. Even though the input mask and calculation are currently limited to members, the tool’s availability can be extended to other countries as well. The tool depends on the availability of input data only and is not limited to a country or region. With the development of more precise processing and classification of Earth observation data, the availability of such data will be possible in the future.

## 5. Conclusions

We developed a new web tool that allows better planning of crop rotation distance to last year’s pea sites by estimating the risk of infestation of mono- or oligophagous insect pests, who overwinter site-specifically. We recommend cultivating peas outside the risky areas, which is at least 1261 to 1825 m from last year’s pea sites, depending on the cultivation type. Currently, it is available throughout Germany, though use may be extended in the future to other countries as well. If the land use data are available, the tool is very convenient to plan crop rotation distance. This may lead to a reduced application of insecticides [11]. A major advantage of this tool is its adaptability to other pests and crops.

## Figures and Tables

**Table 1:** Number of sample sites per federal state in Germany, separated by with/without insecticides.

	Federal State/Year	ST	SN	HE	Federal State/Year		SN
Grain peas	2016	57/1	7/21	3/31	Green peas	2016	33/19
	2017	50/3	4/15	14/44		2017	13/24
	2018	32/9	2/9	11/34		2018	20/32
	2019		2/6	0/15		2019	0/2
	Subtotal	139/13	15/51	28/124			66/77
	Total	152	66	152			143

**Table 2:** Parameters and estimates of the infestation–distance–correlation for grain peas, the significance of the parameters is as follows: <0.001 = ***, <0.01 = **; SE = standard error.

	Parameter	a	b	RMSE	R^2^_adjusted_
All grain peas	Estimate	9.79 ***	1260.70 ***	9.10	0.05
	SE	0.92	299.45		
Without insecticide treatment	Estimate	12.85 ***	1426.23 **	9.98	0.08
	SE	1.45	398.92		
With insecticide treatment	Estimate	8.13 ***	619.05 **	7.41	0.07
	SE	1.09	187.02		

## Data Availability

The data presented in this study are available on request from the corresponding author.
